# Complete Genome Sequence of the Biocontrol Agent *Bacillus velezensis* UFLA258 and Its Comparison with Related Species: Diversity within the Commons

**DOI:** 10.1093/gbe/evz208

**Published:** 2019-10-03

**Authors:** Fabíola de Jesus Silva, Larissa Carvalho Ferreira, Vicente Paulo Campos, Valter Cruz-Magalhães, Aline Ferreira Barros, Jackeline Pereira Andrade, Daniel P Roberts, Jorge Teodoro de Souza

**Affiliations:** 1 Phytopathology Department, Federal University of Lavras, Brazil; 2 Plant Pathology Department, Aberystwyth University, United Kingdom; 3 Biological Sciences Department, Feira de Santana State University, Brazil; 4 USDA-Agricultural Research Service, Sustainable Agricultural Systems Laboratory, Beltsville, Maryland

**Keywords:** biological control, *Bacillus* spp, comparative genomics, CRISPr/Cas, secondary metabolites

## Abstract

In this study, the full genome sequence of *Bacillus velezensis* strain UFLA258, a biological control agent of plant pathogens was obtained, assembled, and annotated. With a comparative genomics approach, in silico analyses of all complete genomes of *B. velezensis* and closely related species available in the database were performed. The genome of *B. velezensis* UFLA258 consisted of a single circular chromosome of 3.95 Mb in length, with a mean GC content of 46.69%. It contained 3,949 genes encoding proteins and 27 RNA genes. Analyses based on Average Nucleotide Identity and Digital DNA–DNA Hybridization and a phylogeny with complete sequences of the *rpo*B gene confirmed that 19 strains deposited in the database as *Bacillus amyloliquefaciens* were in fact *B. velezensis*. In total, 115 genomes were analyzed and taxonomically classified as follows: 105 were *B. velezensis*, 9 were *B. amyloliquefaciens*, and 1 was *Bacillus siamensis*. Although these species are phylogenetically close, the combined analyses of several genomic characteristics, such as the presence of biosynthetic genes encoding secondary metabolites, CRISPr/Cas arrays, Average Nucleotide Identity and Digital DNA–DNA Hybridization, and other information on the strains, including isolation source, allowed their unequivocal classification. This genomic analysis expands our knowledge about the closely related species, *B. velezensis*, *B. amyloliquefaciens*, and *B. siamensis*, with emphasis on their taxonomical status.

## Introduction

Numerous microorganisms have been successfully developed as biopesticides at the commercial level (Shafi et al. 2017). Members of the genus *Bacillus* have been used for this purpose due to their ability to produce a large number of biologically active molecules with growth*-*promoting activity and inhibitory effects against plant pathogens (Fira et al. 2018; Jiang et al. 2018; Olishevska et al. 2019). The potential of *Bacillus* isolates for commercial development is enhanced by their fast growth rate and resistance to adverse environmental conditions (Shafi et al. 2017).

*Bacillus velezensis* was originally described in 2005 (Ruiz-García et al. 2005), and since then its biopesticide potential has been unequivocally shown (Cai et al. 2017; Gao et al. 2017; Jiang et al. 2018). This species synthesizes several types of lipopeptides as products of secondary metabolism. Some of these compounds are active against plant pathogens and/or induce systemic resistance in plants, conferring an adaptive advantage in specific ecological niches (Mukherjee and Das 2005; Ruiz-García et al. 2005; Yamamoto et al. 2015; Lim et al. 2017).

Initially, *B. velezensis* was shown to be closely related to *Bacillussubtilis* and *Bacillusamyloliquefaciens* (Ruiz-García et al. 2005). Subsequently, *B. velezensis* was found to be a heterotypic synonym of *B. amyloliquefaciens* subsp. *plantarum*, *Bacillusmethylotrophicus*, and *Bacillusoryzicola* (Wang et al. 2008; Dunlap et al. 2016). Although all these species were reclassified as *B. velezensis*, this information still needs to be integrated into a well-organized resource.

Strain UFLA258 of *B. velezensis* was isolated from soil around the roots of healthy cotton plants and shown to have potential to control plant pathogens (Medeiros et al. 2011, 2015; Martins et al. 2013, 2018, 2019). In this study, we sequenced the genome of strain UFLA258 and compared it with all genomes of closely related species. Additionally, a taxonomic re-evaluation of the clade *B. velezensis*–*B. amyloliquefaciens* was performed. Although closely related, they are distinct species with many commonalities and minor differences.

## Materials and Methods

### Isolation and DNA Extraction

*Bacillus velezensis* UFLA258 was isolated from a soil sample collected in a cotton (*Gossypium hirsutum* L.) field in Mato Grosso province, Brazil. DNA extraction was done according to the method described by Lee et al. (2003).

### Genome Sequencing and Assembly

The sequence data were generated with an Illumina NextSeq-500 using the run kit Illumina NextSeq 500/550 High Output Kit v2. Sequencing resulted in 22,196,922 reads, with length varying from 32 to 151 bases, which comprised a total of 3,351,735, 222 bases and represented 849-fold genome coverage. The quality was checked with the program FastQC v0.11.5 (Andrews 2010). The genome was assembled employing the assembly service “auto” available in PATRIC (Pathosystems Resource Integration Center; Wattam et al. 2014). This strategy implements BayesHammer (Nikolenko et al. 2013) in short reads, followed by three assembly strategies that include Velvet (Zerbino and Birney 2008), IDBA 1.1.1 (Peng et al. 2010), and SPAdes 3.10.0 (Bankevich et al. 2012). Based on each assembly score provided by the QUAST (Quality Assessment Tool for Genome Assemblies) algorithm (Gurevich et al. 2013), the SPAdes assembly was chosen to move on to the subsequent steps. The 1,304 contigs generated were united into 12 scaffolds using the CONTIGuator web server (Galardini et al. 2011) with *B. velezensis* strain UCMB5113 (accession number NC_022081.1) as the reference genome. The gene *dnaA* was determined as the beginning of the chromosome using an in-house script. Finally, gaps were closed by a de novo strategy with FGAP (Piro et al. 2014), and by reference using NCBI’s BlastN (Altschul et al. 1990) and read mapping in CLC Genomics Workbench 11 (Qiagen Inc.), resulting in a completely closed circle.

### Genome Annotation and Manual Curation

The genome of strain UFLA258 was annotated using the RASTtk (Rapid Annotation Using Subsystem Technology; Brettin et al. 2015) annotation service in PATRIC. Manual curation was conducted through Artemis 16.0.0 software (Rutherford et al. 2000) and insertion/deletion (indels) errors were corrected in CLC Genomics Workbench 11 and adjusted, as there was enough depth coverage. Genes with potential frameshifts were compared with other complete genes with BlastN against the NR database at NCBI and adjusted when necessary. Translated protein sequences were determined with BlastP against the UniProt database (UniProt Consortium 2017). Ribosomal RNA genes were detected using the web-tool RNAmmer 1.2 (Lagesen et al. 2007) and tRNA genes were verified with tRNAscan-SE 2.0 (Lowe and Chan 2016). Clusters of orthologous groups (COGs) were defined with the eggNOG v. 4.5.1 database (Huerta-Cepas et al. 2016).

### Comparative Genomics

All complete genome sequences of *B. velezensis*, *B. amyloliquefaciens*, and *Bacillus siamensis* strains available in the GenBank database (https://www.ncbi.nlm.nih.gov) as of June 24, 2019 were used in this study. Digital DNA–DNA Hybridization (dDDH) and Average Nucleotide Identity (ANI) comparisons were calculated using JspeciesWS (Richter et al. 2016) and Kostas Lab (Rodriguez-R and Konstantinidis 2014), respectively. The genome sequence of the type strain *B. velezensis* FZB42 was used as a reference (accession number NC_009725.1). CRISPr (clustered regularly interspaced short palindromic repeat) matrices and phages were identified using the web-tool CRISPRfinderCAS (Couvin et al. 2018) and PHASTER (Arndt et al. 2016), respectively. Clusters of biosynthetic genes from secondary metabolites were predicted using antiSMASH 4.0.2 (Weber et al. 2015). Principal component analysis (PCA) was performed with the comparative genomics data with packages cluster and factoextra implemented in the R software (R Core Team 2019).

### Phylogenetic Analyses

Complete *rpoB* gene (β subunit of the RNA polymerase) sequences were retrieved from the genomes under study and used for the phylogenetic analysis. Alignments were performed with MAFFT v7.0 (Katoh et al. 2017). A maximum likelihood tree with the T92 + G + I model was constructed using MEGA v10.1 (Kumar et al. 2018) with 1,000 bootstrap replicates.

## Results and Discussion

### Properties of the Genome of *B. velezensis* UFLA258

**Fig. 1.**
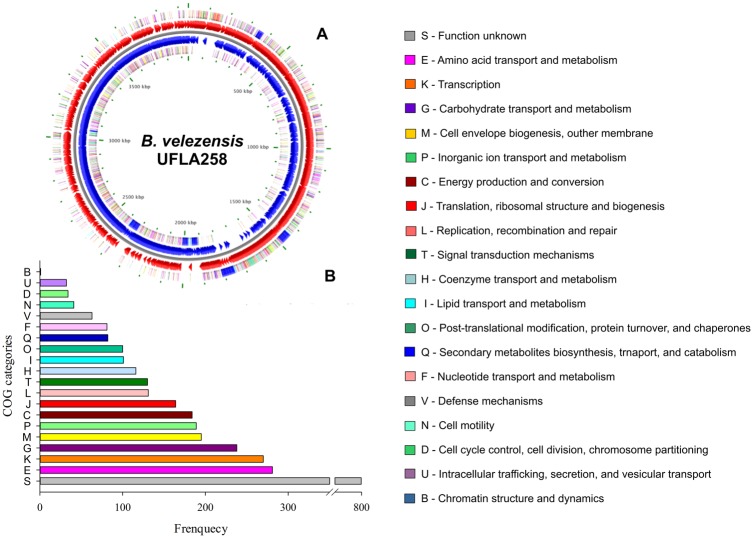
—(*A*) Graphical circular map of *Bacillus velezensis* strain UFLA258 chromosome. From outer circle to the center: CDS on forward strand (colored according to COG categories), all CDS and RNA genes on forward strand, all CDS and RNA genes on reverse strand, CDS on reverse strand (colored according to COG categories). The map was generated using Bacterial Annotation System, BASys (Van Domselaar et al. 2005). (*B*) COG functional classification of the 3,439 proteins.

The genome of *B. velezensis* UFLA258 is composed of an unique circular chromosome of 3.95 Mb (fig. 1), which falls between 3.71 and 4.39 Mb, the size range reported for this species (table 1). The chromosome is predicted to include 3,949 protein-encoding genes, from which 3,747 genes were functionally assigned, whereas the remaining genes were annotated as hypothetical proteins. Pseudogenes accounted for 1.7% of the total number of genes. There were 84 tRNA genes and 9 copies of the ribosomal RNA operon distributed throughout the genome, which represented 27 rRNA genes (table 1). From the predicted genes, 3,439 (87.08%) were classified into 20 functional COG categories, whereas the remaining 510 (12.92%) were not classified into COGs (fig. 1). The most numerous COGs contained genes with unknown function (806 genes), genes involved in the transport and metabolism of amino acids (281 genes) and genes involved in transcription (270 genes). COG categories with the lowest number of genes were genes related to chromatin dynamics and structure and a gene for RNA processing (fig. 1).

**Table 1:** Assembly Statistics and Genome Features of *Bacillus velezensis* Strain UFLA258

Attribute	Value
**(a) Genome statistics**	
Contigs	55
Largest contig (bp)	999,521
Total length (bp)	3,947,620
N50	623,714
L50	3
L75	4
GC (%)	46.5
**(b) Genome features**	
Chromosome size (bp)	3,947,206
Chromosomal genes (number)	3,929
Protein coding genes	3,747
RNA genes	116
Pseudogenes	66
Genes with function prediction	3,439
CRISPR arrays	1

### Phylogeny and Species Boundaries in the Clade *B. amyloliquefaciens*–*B. velezensis*

The complete genome of strain UFLA258 and genomes of 86 strains of *B. velezensis*, 28 of *B. amyloliquefaciens*, and 1 of *B. siamensis* available in the NCBI database were used in the analyses. Sequences of the 16S rRNA of strain UFLA258 were respectively 99.9%, 99.8%, and 99.5% identical to the 16S sequences of the type strains of *B. velezensis*, *B. siamensis*, and *B. amyloliquefaciens*. The sequence of the *rpo*B gene of strain UFLA258 was 99.7% identical to the same sequence of the type strain of *B. velezensis* (FZB42) and 98.9% and 98.5% identical, respectively when compared with the type strains of *B. amyloliquefaciens* (DSM7) and *B. siamensis* (SCSIO 04756). According to the dDDH and ANI values (supplementary tables S1 and S2, Supplementary Material online) and the phylogenetic analyses with the *rpoB* gene (supplementary fig. S1, Supplementary Material online), strain UFLA258 and 104 additional strains belonged in the species *B. velezensis*, whereas 9 other strains were *B. amyloliquefaciens* and 1 was *B. siamensis*. ANI and dDDH values were above the cutoff for the delimitation of each species (ANI > 95%, Auch et al. 2010; dDDH > 70%, Richter et al. 2016).

The phylogenetic analysis corroborated the ANI and dDDH results, showing that most strains identified as *B. amyloliquefaciens* were indeed *B. velezensis* (supplementary fig. S1, Supplementary Material online). The resolution power of the *rpoB* gene in phylogenetic analyses has been shown by several authors (Sharma and Patil 2011; Fan et al. 2017). Recently, the designation “operational group *B. amyloliquefaciens*” has been proposed to name the closely related species *B. amyloliquefaciens*, *B. siamensis*, and *B. velezensis* (Fan et al. 2017). However, we propose the identification by species names as it is easy enough to perform by the methods described above, including *rpoB* phylogeny and genomic indices (ANI and dDDH). The use of individual species names will facilitate scientific communication. Additionally, the species *B. amyloliquefaciens* is less frequently encountered than *B. velezensis*.

### Comparison of UFLA258 with Genomes of Related Species

All deposited genomes of *B. velezensis* were used in this part of the analysis, including genomes of reidentified strains, totaling 105 genomes. Among the complete genomes of *B. velezensis*, the number of genes ranged from 3, 683 to 4, 744 and the number for *B. velezensis* UFLA258 fits within this range (supplementary table S1, Supplementary Material online). Similarly, the GC content (46.69%) and mean size of the genome (4.03 Mb) of *B. velezensis* UFLA258 were comparable to deposited genomes of other *B. velezensis* strains (supplementary table S1, Supplementary Material online).

The genomes of *B. velezensis* encoded 12 groups of genes involved in the production of antimicrobial compounds (supplementary table S3, Supplementary Material online). Among these, five groups of nonribosomal peptide synthase genes, including bacilysin, bacillibactin, fengyncin, bacillaene, and surfactin biosynthesis, were absolutely conserved in all 105 *B. velezensis* genomes used in this study, whereas the polyketide synthase (PKS) genes for difficidin and macrolactin biosynthesis were not present in one strain, AGVL-005 (supplementary table S3, Supplementary Material online). Genes for the compounds plantathizolicin, mersacidin, subtilin, bacillomycin, and locilomycin showed a more variable pattern and occurred in 18, 6, 5, 1, and 1 genomes, respectively. Genomes of *B. amyloliquefaciens* generally did not harbor polyketide synthase genes, except for two strains that encoded macrolactin. Another difference was the absence of the compounds described above as having a variable pattern of occurrence in genomes of *B. velezensis* (supplementary table S3, Supplementary Material online). However, these differences may not be consistent among *B. amyloliquefaciens* due to the low number of genomes of this species available. Most of the abovementioned compounds have surfactant and antibiotic activities and were shown to be active against plant pathogens (Scholz et al. 2011, 2014; Sumi et al. 2015).

The PCA performed with strains from different continents, isolation sources, ANI, dDDH, secondary metabolite profile, among others, revealed that 103 out of the 105 strains of *B. velezensis* grouped closer, whereas the 2 remaining strains were separated from this main group (supplementary fig. S2*A*, Supplementary Material online). Strain OSY-S3 of *B. velezensis* separated from the others due to the presence of a greater number of CRISPr arrays in its genome and the presence of genes encoding the compounds bacilomycin and plantathiazolincin, whereas strain AGVL-005 lacked difficidin and macrolactin genes, which is also the case for genomes of *B. amyloliquefaciens*. Strains of *B. amyloliquefaciens* and the only *B. siamensis* strain available clustered apart from each other and from *B. velezensis* (supplementary fig. S2*A*, Supplementary Material online).

The secondary metabolites difficidin and macrolactin, along with ANI and dDDH, were the variables that contributed the most in the PCA (supplementary fig. S2*B*, Supplementary Material online). Despite the fact that *B. velezensis* represents a globally distributed species, 75% of the strains deposited in the database were from the Asian continent, mostly from China. The others were from the American (14.3%) and European (9.5%) continents, and only one was from Africa. Most *B. velezensis* strains were obtained from rhizosphere/plant (40%), soil (30%), and food (22%). Within *B. amyloliquefaciens*, most isolates were also Asian, but with the predominance of strains isolated from food (supplementary table S3, Supplementary Material online).

CRISPr/Cas arrays are part of the immune system of bacteria and archaea (Cain and Boinett 2013), protecting them against invaders such as bacteriophages and plasmids (Rath et al. 2015). These arrays were found in more than 85% of the genomes of *B. velezensis* studied. When present, the number of Cas copies was always higher than the number of CRISPr copies, with the exception of strain OSY-S3, with five copies of each. On the other hand, only 33% of the genomes of *B. amyloliquefaciens* possessed CRISPr/Cas arrays. However, due to the low number of genomes of this species, little can be inferred at this time. CRISPr loci may be used to provide phylogenetic relationships among bacterial lineages and more recently have been used as tools for transformation (Cain and Boinett 2013; Supply et al. 2013; Altenbuchner 2016).

This comparative analysis provided a comprehensive understanding of the genomes of *B. velezensis* and closely related species. Special emphasis was given to the taxonomic classification of this group, where genomes of 115 strains were evaluated. From these, a total of 19 strains deposited as *B. amyloliquefaciens* were reclassified as *B. velezensis*. In summary, 105 strains were shown to be *B. velezensis*, 9 were *B. amyloliquefaciens*, and 1 was *B. siamensis* (supplementary table S1, Supplementary Material online).

## Supplementary Material

Supplementary data are available at *Genome Biology and Evolution* online.

## Supplementary Material

evz208_Supplementary_Data

## Notes

**Data deposition**: Trimmed sequence data and assembly are deposited in GenBank (accession number: NZ_CP039297.1).
