# Supplementary material for: Complete Genome Sequence of the Biocontrol Agent Bacillus velezensis UFLA258 and Its Comparison with Related Species: Diversity within the Commons
Source: Genome Biol Evol. 2019 Oct 3;11(10):2818–23. doi: 10.1093/gbe/evz208 (PMC6788494; doi:10.1093/gbe/evz208)
Supplement: evz208_Supplementary_Data [file evz208_supplementary_data.zip › Supplementary Material unmarked.docx]

**Supplementary Material**

**Figure S1**. Phylogenetic tree with the taxonomic placement of strain UFLA258. The phylogenetic tree was constructed in MEGA X v10.1 (Kumar et al. 2018) based on complete nucleotide sequences of the *rpoB* gene (3,582 bp) aligned in MAFFT (Katoh et al. 2017). The tree was constructed with the Maximum Likeliood method and the Kimura 2-parameter model. Bootstrap values were calculated with 1,000 resamplings. The red arrow points out strain UFLA258, which had its genome newly sequenced. The scale indicates the number of substitutions per site.

**Figure S2**. Principal components analysis (PCA) of 104 strains of *Bacillus velezensis* performed based on ANI, dDDH, secondary metabolite profiles, origin, source, presence of CRISPr/Cas arrays and phages. A) Clustering of the 104 strains of *B. velezensis* and 9 strains of *B. amyloliquefaciens*, including the strain type DSM 7. B) Variables used in the PCA colored according to their relative contribution rates to the analysis.

**Table S1**. Species boundaries for *Bacillus velezensis*, *B. amyloliquefaciens* and *B. siamensis* based on genomic properties and indexes.

| **Strain** | **Size (Mb)** | **GC (%)** |  | **Protein** | **ANI (%)** | **dDDH (%)** |  |  |
| --- | --- | --- | --- | --- | --- | --- | --- | --- |
| ***B. velezensis*** | | | | | | | |  |
| UFLA258 | 3.95 | 46.69 |  | 3,147 | 98.87 | 92.00 |  |  |
| Other 104 strains | 3.68-4.39 | 43.23-47.00 |  | 2,251-4,185 | 97.57-98.89 | 80.10-92.00 |  |  |
| ***B. amyloliquefaciens*** | | | | | | | |  |
| 9 strains | 3.68-4.08 | 45.69-46.30 |  | 3,501-4,028 | 97.88-99.98 | 81.30-100.0 |  |  |
| ***B. siamensis*** | | | | | | | |  |
| 1 strain | 4.28 | 45.97 |  | 4,150 | 100.00 | 100.00 |  |  |
|  | | | | | | | | |

**Table S2**. Genomes used in this study with the taxonomical re-identification of some *B. amyloliqefaciens* based on ANI and dDDH values.

| **Strains** | **Accession** | | **Current classification** | | **FZB42^T^** | | **DSM 7^T^** | | **Re-identification** |
| --- | --- | --- | --- | --- | --- | --- | --- | --- | --- |
|  |  |  |  |  | **ANI (%)** | **dDDH (%)** | **ANI (%)** | **dDDH (%)** |  |
| 83 | | NZ_CP034203.1 | *B. velezensis* | 98.15 | | 85.80 | **-** | **-** | = |
| 157 | | NZ_CP022341.1 | *B. velezensis* | 98.21 | | 85.80 | **-** | **-** | = |
| 10075 | | NZ_CP025939.1 | *B. velezensis* | 97.91 | | 85.30 | **-** | **-** | = |
| [8_2](https://www.ncbi.nlm.nih.gov/genome/30950?genome_assembly_id=371803) | | NZ_CP028439.1 | *B. velezensis* | 97.64 | | 80.60 | **-** | **-** | = |
| [131-4](https://www.ncbi.nlm.nih.gov/genome/30950?genome_assembly_id=371804) | | NZ_CP028441.1 | *B. velezensis* | 97.62 | | 80.60 | **-** | **-** | = |
| [1B-23](https://www.ncbi.nlm.nih.gov/genome/30950?genome_assembly_id=426189) | | NZ_CP033967.1 | *B. velezensis* | 98.55 | | 91.10 | **-** | **-** | = |
| [9912D](https://www.ncbi.nlm.nih.gov/genome/30950?genome_assembly_id=291145) | | NZ_CP017775.1 | *B. velezensis* | 97.77 | | 83.20 | **-** | **-** | = |
| [9D-6](https://www.ncbi.nlm.nih.gov/genome/30950?genome_assembly_id=315370) | | NZ_CP020805.1 | *B. velezensis* | 98.74 | | 91.20 | **-** | **-** | = |
| [AGVL-005](https://www.ncbi.nlm.nih.gov/genome/30950?genome_assembly_id=354824) | | CP024922.1 | *B. velezensis* | 98.30 | | 89.70 | **-** | **-** | = |
| ANSB01E | | [NZ_CP036518.1](https://www.ncbi.nlm.nih.gov/nuccore/NZ_CP036518.1) | *B. velezensis* | 98.11 | | 85.30 | **-** | **-** | = |
| [AS43.3](https://www.ncbi.nlm.nih.gov/genome/30950?genome_assembly_id=247087) | | NC_019842.1 | *B. velezensis* | 98.74 | | 91.40 | **-** | **-** | = |
| [ATR2](https://www.ncbi.nlm.nih.gov/genome/30950?genome_assembly_id=399491) | | NZ_CP018133.1 | *B. velezensis* | 98.01 | | 85.60 | **-** | **-** | = |
| B25 | | NZ_LN999829.1 | *B. velezensis* | 97.60 | | 80.40 | **-** | **-** | = |
| [Bac57](https://www.ncbi.nlm.nih.gov/genome/30950?genome_assembly_id=412447) | | NZ_CP033054.1 | *B. velezensis* | 97.79 | | 84.40 | **-** | **-** | = |
| [BCSo1](https://www.ncbi.nlm.nih.gov/genome/30950?genome_assembly_id=426635) | | NZ_CP034037.1 | *B. velezensis* | 98.89 | | 90.70 | **-** | **-** | = |
| [BIM B-439D](https://www.ncbi.nlm.nih.gov/genome/30950?genome_assembly_id=405781) | | NZ_CP032144.1 | *B. velezensis* | 98.74 | | 90.40 | **-** | **-** | = |
| [BS-37](https://www.ncbi.nlm.nih.gov/genome/30950?genome_assembly_id=373695) | | NZ_CP023414.1 | *B. velezensis* | 98.68 | | 90.40 | **-** | **-** | = |
| [CAU B946](https://www.ncbi.nlm.nih.gov/genome/30950?genome_assembly_id=247085) | | NC_016784.1 | *B. velezensis* | 97.51 | | 80.20 | **-** | **-** | = |
| [CBMB205](https://www.ncbi.nlm.nih.gov/genome/30950?genome_assembly_id=316469) | | NZ_CP011937.1 | *B. velezensis* | 98.12 | | 85.40 | **-** | **-** | = |
| [CBMB205](https://www.ncbi.nlm.nih.gov/genome/30950?genome_assembly_id=399492) | | NZ_CP014838.1 | *B. velezensis* | 98.12 | | 85.40 | **-** | **-** | = |
| [CC09](https://www.ncbi.nlm.nih.gov/genome/30950?genome_assembly_id=276551) | | NZ_CP015443.1 | *B. velezensis* | 98.38 | | 88.90 | **-** | **-** | = |
| CGMCC11640 | | NZ_CP026610.1 | *B. velezensis* | 98.26 | | 88.90 | **-** | **-** | = |
| [CMT-6](https://www.ncbi.nlm.nih.gov/genome/30950?genome_assembly_id=358072) | | NZ_CP025341.1 | *B. velezensis* | 97.54 | | 81.00 | **-** | **-** | = |
| [CN026](https://www.ncbi.nlm.nih.gov/genome/30950?genome_assembly_id=354825) | | NZ_CP024897.1 | *B. velezensis* | 98.15 | | 85.60 | **-** | **-** | = |
| [DKU_NT_04](https://www.ncbi.nlm.nih.gov/genome/30950?genome_assembly_id=364621) | | NZ_CP026533.1 | *B. velezensis* | 97.71 | | 82.90 | **-** | **-** | = |
| [DR-08](https://www.ncbi.nlm.nih.gov/genome/30950?genome_assembly_id=371736) | | NZ_CP028437.1 | *B. velezensis* | 98.13 | | 85.40 | **-** | **-** | = |
| [DSYZ](https://www.ncbi.nlm.nih.gov/genome/30950?genome_assembly_id=385418) | | NZ_CP030150.1 | *B. velezensis* | 98.25 | | 88.70 | **-** | **-** | = |
| FZB42 | | NC_009725.1 | *B. velezensis* | 0.00 | | 100.00 | **-** | **-** | = |
| [G341](https://www.ncbi.nlm.nih.gov/genome/30950?genome_assembly_id=272531) | | NZ_CP011686.1 | *B. velezensis* | 98.73 | | 90.40 | **-** | **-** | = |
| [GFP-2](https://www.ncbi.nlm.nih.gov/genome/30950?genome_assembly_id=358812) | | NZ_CP021011.1 | *B. velezensis* | 97.71 | | 80.80 | **-** | **-** | = |
| [GH1-13](https://www.ncbi.nlm.nih.gov/genome/30950?genome_assembly_id=307900) | | NZ_CP019040.1 | *B. velezensis* | 97.43 | | 80.80 | **-** | **-** | = |
| [GQJK49](https://www.ncbi.nlm.nih.gov/genome/30950?genome_assembly_id=321406) | | NZ_CP021495.1 | *B. velezensis* | 98.10 | | 85.40 | **-** | **-** | = |
| [GYL4](https://www.ncbi.nlm.nih.gov/genome/30950?genome_assembly_id=373362) | | NZ_CP020874.1 | *B. velezensis* | 98.12 | | 86.80 | **-** | **-** | = |
| [Hx05](https://www.ncbi.nlm.nih.gov/genome/30950?genome_assembly_id=408305) | | NZ_CP029473.1 | *B. velezensis* | 97.53 | | 80.10 | **-** | **-** | = |
| [J7-1](https://www.ncbi.nlm.nih.gov/genome/30950?genome_assembly_id=371802) | | NZ_CP028440.1 | *B. velezensis* | 97.59 | | 80.60 | **-** | **-** | = |
| [JJ-D34](https://www.ncbi.nlm.nih.gov/genome/30950?genome_assembly_id=230419) | | NZ_CP011346.1 | *B. velezensis* | 97.50 | | 80.30 | **-** | **-** | = |
| [JS25R](https://www.ncbi.nlm.nih.gov/genome/30950?genome_assembly_id=212217) | | NZ_CP009679.1 | *B. velezensis* | 98.19 | | 85.80 | **-** | **-** | = |
| [JT3-1](https://www.ncbi.nlm.nih.gov/genome/30950?genome_assembly_id=409223) | | NZ_CP032506.1 | *B. velezensis* | 98.09 | | 85.40 | **-** | **-** | = |
| [JTYP2](https://www.ncbi.nlm.nih.gov/genome/30950?genome_assembly_id=312586) | | NZ_CP020375.1 | *B. velezensis* | 98.09 | | 85.40 | **-** | **-** | = |
| [K26](https://www.ncbi.nlm.nih.gov/genome/30950?genome_assembly_id=399493) | | NZ_CP023075.1 | *B. velezensis* | 97.87 | | 84.40 | **-** | **-** | = |
| [KD1](https://www.ncbi.nlm.nih.gov/genome/30950?genome_assembly_id=379006) | | NZ_CP014990.2 | *B. velezensis* | 97.71 | | 81.30 | **-** | **-** | = |
| [L-1](https://www.ncbi.nlm.nih.gov/genome/30950?genome_assembly_id=349139) | | NZ_CP023859.1 | *B. velezensis* | 98.71 | | 91.10 | **-** | **-** | = |
| LAMBIM40 | | [NZ_CP023748.1](https://www.ncbi.nlm.nih.gov/nuccore/NZ_CP023748.1) | *B. velezensis* | 98.72 | | 91.00 | **-** | **-** | = |
| LB002 | | [NZ_CP037417.1](https://www.ncbi.nlm.nih.gov/nuccore/NZ_CP037417.1) | *B. velezensis* | 97.36 | | 80.40 | **-** | **-** | = |
| [LDO2](https://www.ncbi.nlm.nih.gov/genome/30950?genome_assembly_id=373694) | | NZ_CP029034.1 | *B. velezensis* | 98.10 | | 85.40 | **-** | **-** | = |
| [L-H15](https://www.ncbi.nlm.nih.gov/genome/30950?genome_assembly_id=426906) | | NZ_CP010556.1 | *B. velezensis* | 97.62 | | 80.20 | **-** | **-** | = |
| [LPL-K103](https://www.ncbi.nlm.nih.gov/genome/30950?genome_assembly_id=500087) | | NZ_CP039380.1 | *B. velezensis* | 98.45 | | 89.90 | **-** | **-** | = |
| LS69 | | NZ_CP0159111 | *B. velezensis* | 98.34 | | 85.30 | **-** | **-** | = |
| [L-S60](https://www.ncbi.nlm.nih.gov/genome/30950?genome_assembly_id=426907) | | NZ_CP011278.1 | *B. velezensis* | 97.62 | | 80.30 | **-** | **-** | = |
| [Lzh-a42](https://www.ncbi.nlm.nih.gov/genome/30950?genome_assembly_id=358025) | | NZ_CP025308.1 | *B. velezensis* | 98.10 | | 85.60 | **-** | **-** | = |
| [M75](https://www.ncbi.nlm.nih.gov/genome/30950?genome_assembly_id=282030) | | NZ_CP016395.1 | *B. velezensis* | 97.58 | | 80.60 | **-** | **-** | = |
| [MH25](https://www.ncbi.nlm.nih.gov/genome/30950?genome_assembly_id=426908) | | NZ_CP034176.1 | *B. velezensis* | 98.71 | | 91.10 | **-** | **-** | = |
| [NAU-B3](https://www.ncbi.nlm.nih.gov/genome/30950?genome_assembly_id=247091) | | NC_022530.1 | *B. velezensis* | 98.09 | | 85.80 | **-** | **-** | = |
| [NJAU-Z9](https://www.ncbi.nlm.nih.gov/genome/30950?genome_assembly_id=325072) | | NZ_CP022556.1 | *B. velezensis* | 97.62 | | 80.70 | **-** | **-** | = |
| [NJN-6](https://www.ncbi.nlm.nih.gov/genome/30950?genome_assembly_id=247094) | | NZ_CP007165.1 | *B. velezensis* | 97.39 | | 80.50 | **-** | **-** | = |
| [NKG-1](https://www.ncbi.nlm.nih.gov/genome/30950?genome_assembly_id=352107) | | NZ_CP024203.1 | *B. velezensis* | 98.71 | | 92.00 | **-** | **-** | = |
| [NY12-2](https://www.ncbi.nlm.nih.gov/genome/30950?genome_assembly_id=418332) | | NZ_CP033576.1 | *B. velezensis* | 97.79 | | 84.40 | **-** | **-** | = |
| [OSY-S3](https://www.ncbi.nlm.nih.gov/genome/30950?genome_assembly_id=353400) | | CP024706.1 | *B. velezensis* | 98.65 | | 89.60 | **-** | **-** | = |
| [QST713](https://www.ncbi.nlm.nih.gov/genome/30950?genome_assembly_id=373482) | | NZ_CP025079.1 | *B. velezensis* | 98.45 | | 88.30 | **-** | **-** | = |
| [S141](https://www.ncbi.nlm.nih.gov/genome/30950?genome_assembly_id=407009) | | NZ_AP018402.1 | *B. velezensis* | 98.66 | | 89.80 | **-** | **-** | = |
| [S3-1](https://www.ncbi.nlm.nih.gov/genome/30950?genome_assembly_id=279448) | | NZ_CP016371.1 | *B. velezensis* | 98.12 | | 85.40 | **-** | **-** | = |
| [SB1216](https://www.ncbi.nlm.nih.gov/genome/30950?genome_assembly_id=274676) | | CP015417.1 | *B. velezensis* | 98.76 | | 90.20 | **-** | **-** | = |
| [SCDB 291](https://www.ncbi.nlm.nih.gov/genome/30950?genome_assembly_id=329303) | | NZ_CP022654.2 | *B. velezensis* | 97.57 | | 81.30 | **-** | **-** | = |
| [SCGB 1](https://www.ncbi.nlm.nih.gov/genome/30950?genome_assembly_id=329304) | | NZ_CP023320.1 | *B. velezensis* | 97.58 | | 81.20 | **-** | **-** | = |
| [SCGB 574](https://www.ncbi.nlm.nih.gov/genome/30950?genome_assembly_id=330722) | | NZ_CP023431.1 | *B. velezensis* | 98.19 | | 85.90 | **-** | **-** | = |
| [SQR9](https://www.ncbi.nlm.nih.gov/genome/30950?genome_assembly_id=247093) | | NZ_CP006890.1 | *B. velezensis* | 98.06 | | 86.80 | **-** | **-** | = |
| [SRCM100072](https://www.ncbi.nlm.nih.gov/genome/30950?genome_assembly_id=322011) | | NZ_CP021888.1 | *B. velezensis* | 97.75 | | 84.50 | **-** | **-** | = |
| [SRCM101413](https://www.ncbi.nlm.nih.gov/genome/30950?genome_assembly_id=322012) | | NZ_CP021890.1 | *B. velezensis* | 97.82 | | 84.20 | **-** | **-** | = |
| [SRCM103616](https://www.ncbi.nlm.nih.gov/genome/30950?genome_assembly_id=443353) | | NZ_CP035410.1 | *B. velezensis* | 97.83 | | 84.20 | **-** | **-** | = |
| SRCM103691 | | NZ_CP035393.1 | *B. velezensis* | 97.89 | | 84.20 | **-** | **-** | = |
| [SRCM103788](https://www.ncbi.nlm.nih.gov/genome/30950?genome_assembly_id=443352) | | NZ_CP035399.1 | *B. velezensis* | 97.85 | | 84.20 | **-** | **-** | = |
| [sx01604](https://www.ncbi.nlm.nih.gov/genome/30950?genome_assembly_id=312246) | | NZ_CP018007.1 | *B. velezensis* | 98.09 | | 85.40 | **-** | **-** | = |
| [SYBC H47](https://www.ncbi.nlm.nih.gov/genome/30950?genome_assembly_id=290466) | | NZ_CP017747.1 | *B. velezensis* | 97.73 | | 82.00 | **-** | **-** | = |
| [T20E-257](https://www.ncbi.nlm.nih.gov/genome/30950?genome_assembly_id=322115) | | NZ_CP021976.1 | *B. velezensis* | 97.60 | | 80.60 | **-** | **-** | = |
| [TB1501](https://www.ncbi.nlm.nih.gov/genome/30950?genome_assembly_id=324988) | | NZ_CP022531.1 | *B. velezensis* | 98.78 | | 91.10 | **-** | **-** | = |
| [TJ02](https://www.ncbi.nlm.nih.gov/genome/30950?genome_assembly_id=353401) | | NZ_CP024797.1 | *B. velezensis* | 98.74 | | 91.30 | **-** | **-** | = |
| [TrigoCor1448](https://www.ncbi.nlm.nih.gov/genome/30950?genome_assembly_id=247092) | | NZ_CP007244.1 | *B. velezensis* | 98.78 | | 91.60 | **-** | **-** | = |
| [UCMB-5033](https://www.ncbi.nlm.nih.gov/genome/30950?genome_assembly_id=301268) | | NC_022075.1 | *B. velezensis* | 98.65 | | 90.50 | **-** | **-** | = |
| [UCMB5036](https://www.ncbi.nlm.nih.gov/genome/30950?genome_assembly_id=247088) | | NC_020410.1 | *B. velezensis* | 98.55 | | 89.00 | **-** | **-** | = |
| UCMB5113 | | NC_022081.1 | *B. velezensis* | 98.81 | | 90.50 | **-** | **-** | = |
| UFLA258 | | [NZ_CP039297.1](https://www.ncbi.nlm.nih.gov/nuccore/NZ_CP039297.1) | *B. velezensis* | 98.87 | | 92.00 | **-** | **-** | = |
| [W1](https://www.ncbi.nlm.nih.gov/genome/30950?genome_assembly_id=384405) | | NZ_CP028375.1 | *B. velezensis* | 98.15 | | 85.50 | **-** | **-** | = |
| [YAUB9601Y2](https://www.ncbi.nlm.nih.gov/genome/30950?genome_assembly_id=247086) | | NC_017061.1 | *B. velezensis* | 98.06 | | 85.70 | **-** | **-** | = |
| YJ11-1-4 | | [NZ_CP011347.1](https://www.ncbi.nlm.nih.gov/nuccore/NZ_CP011347.1) | *B. velezensis* | 98.10 | | 86.80 | **-** | **-** | = |
| [ZF2](https://www.ncbi.nlm.nih.gov/genome/30950?genome_assembly_id=406152) | | NZ_CP032154.1 | *B. velezensis* | 98.13 | | 85.40 | **-** | **-** | = |
| [ZL918](https://www.ncbi.nlm.nih.gov/genome/30950?genome_assembly_id=319055) | | NZ_CP021338.1 | *B. velezensis* | 97.59 | | 80.20 | **-** | **-** | = |
| ALB65 | | NZ_CP029069.1 | *B. amyloliquefaciens* | 98.02 | | 85.30 | **93.39** | **56.10** | *B. velezensis* |
| ALB69 | | NZ_CP029070.1 | *B. amyloliquefaciens* | 98.17 | | 86.40 | **93.35** | **55.80** | *B. velezensis* |
| ALB79 | | NZ_CP029071.1 | *B. amyloliquefaciens* | 98.23 | | 88.50 | **93.29** | **56.00** | *B. velezensis* |
| B15 | | NZ_CP014783.1 | *B. amyloliquefaciens* | 97.56 | | 80.20 | **93.37** | **55.50** | *B. velezensis* |
| B-4 | | NZ_CP031424.1 | *B. amyloliquefaciens* | 98.43 | | 88.60 | **93.4** | **55.60** | *B. velezensis* |
| CC178 | | NC_022653.1 | *B. amyloliquefaciens* | 99.99 | | 100.00 | **93.41** | **56.10** | *B. velezensis* |
| DSM 7 | | NC_014551.1 | *B. amyloliquefaciens* | **93.84** | | **56.20** | 100.00 | 100.00 | *=* |
| FS1092 | | NZ_CP038028.1 | *B. amyloliquefaciens* | 98.14 | | 88.30 | **93.23** | **55.80** | *B. velezensis* |
| HK1 | | NZ_CP018902.1 | *B. amyloliquefaciens* | **93.82** | | **56.10** | 99.98 | 100.00 | *=* |
| IT-45 | | NC_020272.1 | *B. amyloliquefaciens* | 97.6 | | 80.50 | **93.44** | **55.50** | *B. velezensis* |
| KHG19 | | NZ_CP007242.1 | *B. amyloliquefaciens* | 98.74 | | 90.50 | **93.44** | **56.10** | *B. velezensis* |
| LFB112 | | NC_023073.1 | *B. amyloliquefaciens* | 97.45 | | 80.30 | **93.25** | **55.60** | *B. velezensis* |
| LL3 | | NC_017190.1 | *B. amyloliquefaciens* | **93.74** | | **55.70** | 99.47 | 96.40 | *=* |
| LM2303 | | NZ_CP018152.1 | *B. amyloliquefaciens* | 97.53 | | 80.30 | **93.28** | **55.20** | *B. velezensis* |
| MBE1283 | | NZ_CP013727.1 | *B. amyloliquefaciens* | 97.52 | | 79.90 | **93.33** | **55.20** | *B. velezensis* |
| MT45 | | NZ_CP011252.1 | *B. amyloliquefaciens* | **93.92** | | **56.10** | 98.09 | 85.70 | *=* |
| RD7-7 | | NZ_CP016913.1 | *B. amyloliquefaciens* | **93.99** | | **56.30** | 97.57 | 81.30 | *=* |
| S499 | | NZ_CP014700.1 | *B. amyloliquefaciens* | 97.65 | | 80.50 | **93.46** | **55.50** | *B. velezensis* |
| SH-B74 | | NZ_CP030097.1 | *B. amyloliquefaciens* | 98.53 | | 89.90 | **93.31** | **56.10** | *B. velezensis* |
| SRCM101267 | | NZ_CP021505.1 | *B. amyloliquefaciens* | **93.78** | | **56.10** | 99.69 | 98.90 | *=* |
| TA208 | | NC_017188.1 | *B. amyloliquefaciens* | **93.69** | | **55.70** | 99.28 | 95.20 | *=* |
| UMAF6614 | | NZ_CP006960.1 | *B. amyloliquefaciens* | 98.22 | | 86.40 | **93.39** | **56.00** | *B. velezensis* |
| UMAF6639 | | NZ_CP006058.1 | *B. amyloliquefaciens* | 98.21 | | 88.60 | **93.35** | **55.70** | *B. velezensis* |
| WS-8 | | NZ_CP018200.1 | *B. amyloliquefaciens* | 98.13 | | 85.40 | **93.27** | **55.40** | *B. velezensis* |
| XH7 | | NC_017191.1 | *B. amyloliquefaciens* | **93.76** | | **55.70** | 99.31 | 95.40 | *=* |
| Y14 | | NZ_CP017953.1 | *B. amyloliquefaciens* | 98.15 | | 85.60 | **93.25** | **55.90** | *B. velezensis* |
| Y2 | | NC_017912.1 | *B. amyloliquefaciens* | 97.59 | | 80.20 | **93.24** | **55.60** | *B. velezensis* |
| YP6 | | NZ_CP032146.1 | *B. amyloliquefaciens* | **93.90** | | **56.20** | 97.88 | 85.90 | *=* |
| SCSIO 05746 | | [NZ_CP025001.1](https://www.ncbi.nlm.nih.gov/nuccore/NZ_CP025001.1) | *B. siamensis* | **94.15** | | **58.00** | **92.99** | **54.60** | *=* |

Digital DNA-DNA Hybridization (dDDH) and Average Nucleotide Acid Identity (ANI) comparisons with the FZB42 (*B. velezensis* type strain) and DMS 7 (*B. amyloliquefaciens* type strain) genomes. Values out of the range for species delineation are shown in bold (ANI> 95%, Auch et al. 2010; dDDH> 70%, Richter et al. 2015).

**Table S3**. Genomic characteristics, source and origin of the *B. velezensis*, *B. amyloliquefaciens* and *B. siamensis* strains used in this study. These data, together with ANI and dDDH were used in the PCA analysis.

| **Strain** | **Accession** | | **Continent** | **Source** | **CRISPR*** | **Cas*** | **Phage*** | **Metabolites**** |
| --- | --- | --- | --- | --- | --- | --- | --- | --- |
| ***B. velezensis*** | | | | | | | | |
| 83 | NZ_CP034203.1 | | American | Plant | 1 | 8 | 0 |  |
| 157 | NZ_CP022341.1 | | Asian | Plant | 1 | 7 | 1 |  |
| 10075 | NZ_CP025939.1 | | Asian | Food | 2 | 7 | 3 |  |
| [8_2](https://www.ncbi.nlm.nih.gov/genome/30950?genome_assembly_id=371803) | NZ_CP028439.1 | | Asian | Soil | 1 | 6 | 2 |  |
| [131-4](https://www.ncbi.nlm.nih.gov/genome/30950?genome_assembly_id=371804) | NZ_CP028441.1 | | Asian | Soil | 0 | 0 | 2 |  |
| [1B-23](https://www.ncbi.nlm.nih.gov/genome/30950?genome_assembly_id=426189) | NZ_CP033967.1 | | American | Rhizosphere | 0 | 0 | 2 |  |
| [9912D](https://www.ncbi.nlm.nih.gov/genome/30950?genome_assembly_id=291145) | NZ_CP017775.1 | | Asian | Soil | 1 | 7 | 3 |  |
| [9D-6](https://www.ncbi.nlm.nih.gov/genome/30950?genome_assembly_id=315370) | NZ_CP020805.1 | | American | Rhizosphere | 1 | 7 | 0 |  |
| [AGVL-005](https://www.ncbi.nlm.nih.gov/genome/30950?genome_assembly_id=354824) | CP024922.1 | | American | Plant | 0 | 0 | 2 |  |
| ANSB01E | [NZ_CP036518.1](https://www.ncbi.nlm.nih.gov/nuccore/NZ_CP036518.1) | | Asian | Other | 2 | 7 | 1 |  |
| [AS43.3](https://www.ncbi.nlm.nih.gov/genome/30950?genome_assembly_id=247087) | NC_019842.1 | | American | Rhizosphere | 1 | 7 | 1 |  |
| [ATR2](https://www.ncbi.nlm.nih.gov/genome/30950?genome_assembly_id=399491) | NZ_CP018133.1 | | Asian | Soil | 3 | 7 | 1 |  |
| B25 | NZ_LN999829.1 | | Asian | Rhizosphere | 2 | 8 | 0 |  |
| [Bac57](https://www.ncbi.nlm.nih.gov/genome/30950?genome_assembly_id=412447) | NZ_CP033054.1 | | Asian | Soil | 2 | 8 | 1 |  |
| [BCSo1](https://www.ncbi.nlm.nih.gov/genome/30950?genome_assembly_id=426635) | NZ_CP034037.1 | | European | Other | 1 | 7 | 1 |  |
| [BIM B-439D](https://www.ncbi.nlm.nih.gov/genome/30950?genome_assembly_id=405781) | NZ_CP032144.1 | | European | Soil | 3 | 7 | 2 |  |
| [BS-37](https://www.ncbi.nlm.nih.gov/genome/30950?genome_assembly_id=373695) | NZ_CP023414.1 | | Asian | Food | 1 | 7 | 2 |  |
| [CAU B946](https://www.ncbi.nlm.nih.gov/genome/30950?genome_assembly_id=247085) | NC_016784.1 | | Asian | Rhizosphere | 1 | 7 | 1 |  |
| [CBMB205](https://www.ncbi.nlm.nih.gov/genome/30950?genome_assembly_id=316469) | NZ_CP011937.1 | | Asian | Rhizosphere | 2 | 7 | 1 |  |
| [CBMB205](https://www.ncbi.nlm.nih.gov/genome/30950?genome_assembly_id=399492) | NZ_CP014838.1 | | Asian | Rhizosphere | 1 | 7 | 1 |  |
| [CC09](https://www.ncbi.nlm.nih.gov/genome/30950?genome_assembly_id=276551) | NZ_CP015443.1 | | Asian | Plant | 2 | 6 | 1 |  |
| CGMCC11640 | NZ_CP026610.1 | | Asian | Soil | 3 | 6 | 2 |  |
| [CMT-6](https://www.ncbi.nlm.nih.gov/genome/30950?genome_assembly_id=358072) | NZ_CP025341.1 | | Asian | Food | 1 | 7 | 1 |  |
| [CN026](https://www.ncbi.nlm.nih.gov/genome/30950?genome_assembly_id=354825) | NZ_CP024897.1 | | European | Other | 2 | 7 | 0 |  |
| [DKU_NT_04](https://www.ncbi.nlm.nih.gov/genome/30950?genome_assembly_id=364621) | NZ_CP026533.1 | | Asian | Food | 1 | 6 | 1 |  |
| [DR-08](https://www.ncbi.nlm.nih.gov/genome/30950?genome_assembly_id=371736) | NZ_CP028437.1 | | Asian | Soil | 1 | 7 | 1 |  |
| [DSYZ](https://www.ncbi.nlm.nih.gov/genome/30950?genome_assembly_id=385418) | NZ_CP030150.1 | | Asian | Rhizosphere | 3 | 6 | 1 |  |
| FZB42 | NC_009725.1 | | European | Rhizosphere | 0 | 0 | 0 |  |
| [G341](https://www.ncbi.nlm.nih.gov/genome/30950?genome_assembly_id=272531) | NZ_CP011686.1 | | Asian | Rhizosphere | 2 | 7 | 1 |  |
| [GFP-2](https://www.ncbi.nlm.nih.gov/genome/30950?genome_assembly_id=358812) | NZ_CP021011.1 | | Asian | Other | 0 | 0 | 0 |  |
| [GH1-13](https://www.ncbi.nlm.nih.gov/genome/30950?genome_assembly_id=307900) | NZ_CP019040.1 | | Asian | Soil | 2 | 8 | 1 |  |
| [GQJK49](https://www.ncbi.nlm.nih.gov/genome/30950?genome_assembly_id=321406) | NZ_CP021495.1 | | Asian | Rhizosphere | 1 | 7 | 1 |  |
| [GYL4](https://www.ncbi.nlm.nih.gov/genome/30950?genome_assembly_id=373362) | NZ_CP020874.1 | | Asian | Plant | 1 | 7 | 0 |  |
| [Hx05](https://www.ncbi.nlm.nih.gov/genome/30950?genome_assembly_id=408305) | NZ_CP029473.1 | | Asian | Rhizosphere | 0 | 0 | 1 |  |
| [J7-1](https://www.ncbi.nlm.nih.gov/genome/30950?genome_assembly_id=371802) | NZ_CP028440.1 | | Asian | Soil | 1 | 7 | 2 |  |
| [JJ-D34](https://www.ncbi.nlm.nih.gov/genome/30950?genome_assembly_id=230419) | NZ_CP011346.1 | | Asian | Food | 1 | 7 | 1 |  |
| [JS25R](https://www.ncbi.nlm.nih.gov/genome/30950?genome_assembly_id=212217) | NZ_CP009679.1 | | Asian | Plant | 1 | 7 | 1 |  |
| [JT3-1](https://www.ncbi.nlm.nih.gov/genome/30950?genome_assembly_id=409223) | NZ_CP032506.1 | | Asian | Other | 1 | 7 | 0 |  |
| [JTYP2](https://www.ncbi.nlm.nih.gov/genome/30950?genome_assembly_id=312586) | NZ_CP020375.1 | | Asian | Plant | 1 | 7 | 1 |  |
| [K26](https://www.ncbi.nlm.nih.gov/genome/30950?genome_assembly_id=399493) | NZ_CP023075.1 | | Asian | Food | 2 | 7 | 1 |  |
| [KD1](https://www.ncbi.nlm.nih.gov/genome/30950?genome_assembly_id=379006) | NZ_CP014990.2 | | Asian | Food | 0 | 0 | 1 |  |
| [L-1](https://www.ncbi.nlm.nih.gov/genome/30950?genome_assembly_id=349139) | NZ_CP023859.1 | | Asian | Soil | 2 | 6 | 2 |  |
| LAMBIM40 | [NZ_CP023748.1](https://www.ncbi.nlm.nih.gov/nuccore/NZ_CP023748.1) | | American | Faeces | 1 | 7 | 1 |  |
| LB002 | [NZ_CP037417.1](https://www.ncbi.nlm.nih.gov/nuccore/NZ_CP037417.1) | | Asian | Soil | 1 | 7 | 3 |  |
| [LDO2](https://www.ncbi.nlm.nih.gov/genome/30950?genome_assembly_id=373694) | NZ_CP029034.1 | | Asian | Soil | 2 | 7 | 0 |  |
| [L-H15](https://www.ncbi.nlm.nih.gov/genome/30950?genome_assembly_id=426906) | NZ_CP010556.1 | | Asian | Soil | 0 | 0 | 2 |  |
| [LPL-K103](https://www.ncbi.nlm.nih.gov/genome/30950?genome_assembly_id=500087) | NZ_CP039380.1 | | Asian | Plant | 0 | 0 | 1 |  |
| LS69 | NZ_CP0159111 | | Asian | Plant | 1 | 7 | 1 |  |
| [L-S60](https://www.ncbi.nlm.nih.gov/genome/30950?genome_assembly_id=426907) | NZ_CP011278.1 | | Asian | Soil | 0 | 0 | 2 |  |
| [Lzh-a42](https://www.ncbi.nlm.nih.gov/genome/30950?genome_assembly_id=358025) | NZ_CP025308.1 | | Asian | Soil | 2 | 7 | 1 |  |
| [M75](https://www.ncbi.nlm.nih.gov/genome/30950?genome_assembly_id=282030) | NZ_CP016395.1 | | Asian | Soil | 2 | 7 | 2 |  |
| [MH25](https://www.ncbi.nlm.nih.gov/genome/30950?genome_assembly_id=426908) | NZ_CP034176.1 | | Asian | Rhizosphere | 2 | 6 | 3 |  |
| [NAU-B3](https://www.ncbi.nlm.nih.gov/genome/30950?genome_assembly_id=247091) | NC_022530.1 | | Asian | Plant | 1 | 7 | 1 |  |
| [NJAU-Z9](https://www.ncbi.nlm.nih.gov/genome/30950?genome_assembly_id=325072) | NZ_CP022556.1 | | Asian | Soil | 1 | 7 | 1 |  |
| [NJN-6](https://www.ncbi.nlm.nih.gov/genome/30950?genome_assembly_id=247094) | NZ_CP007165.1 | | Asian | Plant | 1 | 7 | 2 |  |
| [NKG-1](https://www.ncbi.nlm.nih.gov/genome/30950?genome_assembly_id=352107) | NZ_CP024203.1 | | Asian | Soil | 0 | 0 | 4 |  |
| [NY12-2](https://www.ncbi.nlm.nih.gov/genome/30950?genome_assembly_id=418332) | NZ_CP033576.1 | | Asian | Food | 2 | 8 | 2 |  |
| [OSY-S3](https://www.ncbi.nlm.nih.gov/genome/30950?genome_assembly_id=353400) | CP024706.1 | | American | Food | 5 | 5 | 2 |  |
| [QST713](https://www.ncbi.nlm.nih.gov/genome/30950?genome_assembly_id=373482) | NZ_CP025079.1 | | European | Soil | 2 | 7 | 2 |  |
| [S141](https://www.ncbi.nlm.nih.gov/genome/30950?genome_assembly_id=407009) | NZ_AP018402.1 | | Asian | Rhizosphere | 1 | 8 | 1 |  |
| [S3-1](https://www.ncbi.nlm.nih.gov/genome/30950?genome_assembly_id=279448) | NZ_CP016371.1 | | Asian | Rhizosphere | 1 | 7 | 1 |  |
| [SB1216](https://www.ncbi.nlm.nih.gov/genome/30950?genome_assembly_id=274676) | CP015417.1 | | American | Soil | 3 | 7 | 2 |  |
| [SCDB 291](https://www.ncbi.nlm.nih.gov/genome/30950?genome_assembly_id=329303) | NZ_CP022654.2 | | Asian | Soil | 1 | 8 | 3 |  |
| [SCGB 1](https://www.ncbi.nlm.nih.gov/genome/30950?genome_assembly_id=329304) | NZ_CP023320.1 | | Asian | Food | 2 | 8 | 1 |  |
| [SCGB 574](https://www.ncbi.nlm.nih.gov/genome/30950?genome_assembly_id=330722) | NZ_CP023431.1 | | Asian | Food | 1 | 7 | 1 |  |
| [SQR9](https://www.ncbi.nlm.nih.gov/genome/30950?genome_assembly_id=247093) | NZ_CP006890.1 | | Asian | Rhizosphere | 0 | 0 | 0 |  |
| [SRCM100072](https://www.ncbi.nlm.nih.gov/genome/30950?genome_assembly_id=322011) | NZ_CP021888.1 | | Asian | Food | 1 | 8 | 1 |  |
| [SRCM101413](https://www.ncbi.nlm.nih.gov/genome/30950?genome_assembly_id=322012) | NZ_CP021890.1 | | Asian | Food | 1 | 6 | 3 |  |
| [SRCM103616](https://www.ncbi.nlm.nih.gov/genome/30950?genome_assembly_id=443353) | NZ_CP035410.1 | | Asian | Food | 1 | 7 | 4 |  |
| SRCM103691 | NZ_CP035393.1 | | Asian | Food | 1 | 7 | 2 |  |
| [SRCM103788](https://www.ncbi.nlm.nih.gov/genome/30950?genome_assembly_id=443352) | NZ_CP035399.1 | | Asian | Food | 1 | 7 | 2 |  |
| [sx01604](https://www.ncbi.nlm.nih.gov/genome/30950?genome_assembly_id=312246) | NZ_CP018007.1 | | Asian | Soil | 2 | 7 | 1 |  |
| [SYBC H47](https://www.ncbi.nlm.nih.gov/genome/30950?genome_assembly_id=290466) | NZ_CP017747.1 | | Asian | Food | 1 | 8 | 1 |  |
| [T20E-257](https://www.ncbi.nlm.nih.gov/genome/30950?genome_assembly_id=322115) | NZ_CP021976.1 | | Asian | Plant | 0 | 0 | 2 |  |
| [TB1501](https://www.ncbi.nlm.nih.gov/genome/30950?genome_assembly_id=324988) | NZ_CP022531.1 | | Asian | Soil | 2 | 6 | 1 |  |
| [TJ02](https://www.ncbi.nlm.nih.gov/genome/30950?genome_assembly_id=353401) | NZ_CP024797.1 | | Asian | Soil | 2 | 7 | 3 |  |
| [TrigoCor1448](https://www.ncbi.nlm.nih.gov/genome/30950?genome_assembly_id=247092) | NZ_CP007244.1 | | American | Rhizosphere | 1 | 8 | 1 |  |
| [UCMB-5033](https://www.ncbi.nlm.nih.gov/genome/30950?genome_assembly_id=301268) | NC_022075.1 | | European | Soil | 1 | 7 | 1 |  |
| [UCMB5036](https://www.ncbi.nlm.nih.gov/genome/30950?genome_assembly_id=247088) | NC_020410.1 | | European | Rhizosphere | 1 | 7 | 1 |  |
| UCMB5113 | NC_022081.1 | | European | Soil | 2 | 6 | 1 |  |
| UFLA258 | [NZ_CP039297.1](https://www.ncbi.nlm.nih.gov/nuccore/NZ_CP039297.1) | | American | Soil | 1 | 7 | 1 |  |
| [W1](https://www.ncbi.nlm.nih.gov/genome/30950?genome_assembly_id=384405) | NZ_CP028375.1 | | Asian | Faeces | 2 | 6 | 0 |  |
| [YAUB9601Y2](https://www.ncbi.nlm.nih.gov/genome/30950?genome_assembly_id=247086) | NC_017061.1 | | Asian | Rhizosphere | 2 | 7 | 0 |  |
| YJ11-1-4 | [NZ_CP011347.1](https://www.ncbi.nlm.nih.gov/nuccore/NZ_CP011347.1) | | Asian | Food | 1 | 7 | 0 |  |
| [ZF2](https://www.ncbi.nlm.nih.gov/genome/30950?genome_assembly_id=406152) | NZ_CP032154.1 | | Asian | Plant | 1 | 7 | 1 |  |
| [ZL918](https://www.ncbi.nlm.nih.gov/genome/30950?genome_assembly_id=319055) | NZ_CP021338.1 | | Asian | Plant | 1 | 7 | 2 |  |
| ALB65 | NZ_CP029069.1 | | American | Food | 1 | 7 | 1 |  |
| ALB69 | NZ_CP029070.1 | | American | Food | 1 | 7 | 2 |  |
| ALB79 | NZ_CP029071.1 | | American | Plant | 2 | 7 | 1 |  |
| B15 | NZ_CP014783.1 | | Asian | Plant | 1 | 8 | 1 |  |
| B-4 | NZ_CP031424.1 | | Asian | Plant | 1 | 8 | 1 |  |
| CC178 | NC_022653.1 | | Asian | Plant | 0 | 0 | 0 |  |
| FS1092 | NZ_CP038028.1 | | American | Food | 2 | 7 | 1 |  |
| IT-45 | NC_020272.1 | | American | Rhizosphere | 2 | 6 | 1 |  |
| KHG19 | NZ_CP007242.1 | | Asian | Food | 2 | 7 | 1 |  |
| LFB112 | NC_023073.1 | | Asian | Other | 2 | 6 | 1 |  |
| LM2303 | NZ_CP007242.1 | | Asian | Faeces | 1 | 7 | 1 |  |
| MBE1283 | NC_023073.1 | | Asian | Food | 1 | 7 | 2 |  |
| S499 | NZ_CP018152.1 | | Africa | Soil | 3 | 7 | 0 |  |
| SH-B74 | NZ_CP013727.1 | | Asian | Soil | 3 | 6 | 1 |  |
| UMAF6614 | NZ_CP014700.1 | | European | Rhizosphere | 1 | 7 | 2 |  |
| UMAF6639 | NZ_CP030097.1 | | European | Rhizosphere | 3 | 7 | 1 |  |
| WS-8 | NZ_CP006960.1 | | Asian | Soil | 2 | 7 | 1 |  |
| Y14 | NZ_CP006058.1 | | Asian | Rhizosphere | 1 | 7 | 1 |  |
| Y2 | NZ_CP018200.1 | | Asian | Rhizosphere | 2 | 7 | 1 |  |
| ***B. amyloliquefaciens*** | | | | | | | | |
| DSM 7 | NC_014551.1 | | European | Soil | 0 | 0 | 5 |  |
| HK1 | NZ_CP018902.1 | | Asian | Other | 0 | 0 | 4 |  |
| LL3 | NC_017190.1 | | Asian | Food | 0 | 0 | 2 |  |
| MT45 | NZ_CP011252.1 | | Asian | Food | 1 | 7 | 1 |  |
| RD7-7 | NZ_CP016913.1 | | Asian | Food | 2 | 8 | 0 |  |
| SRCM101267 | NZ_CP021505.1 | | Asian | Food | 0 | 0 | 4 |  |
| TA208 | NC_017188.1 | | Asian | Food | 0 | 0 | 2 |  |
| XH7 | NC_017191.1 | | Asian | Rhizosphere | 0 | 0 | 2 |  |
| YP6 | NZ_CP032146.1 | | Asian | Rhizosphere | 1 | 7 | 1 |  |
| ***B. siamensis*** | | | | | | | | |
| SCSIO 05746 | [NZ_CP025001.1](https://www.ncbi.nlm.nih.gov/nuccore/NZ_CP025001.1) | | Asian | Soil | 2 | 6 | 2 |  |
| * Number of copies of each element in the genome. | | | | | | | | |
| ** Metabolites: Bacilysin, bacillibactin, difficidin, fengyncin, bacillaen, macrolactin, surfactin, plantathizolicin, subtilin, mersacidin, 000000bacillomycin, locilomycin. | | | | | | | | |
|  | |  | | | | | | |
